# Best evidence summary for self-management of peristomal skin complications in patients with colorectal cancer and a stoma

**DOI:** 10.1016/j.apjon.2025.100822

**Published:** 2025-11-18

**Authors:** Jiayu Qin, Lijun Han, Guifen Lv, Yongmei You, Huiren Zhuang, Lili Ma

**Affiliations:** a Center of Gallbladder Disease, Shanghai East Hospital, Institute of Gallstone Disease, School of Medicine, Tongji University, Shanghai, China; b Colorectal Surgery, Shanghai East Hospital, School of Medicine, Tongji University, Shanghai, China; c Department of Nursing, Shanghai East Hospital, School of Medicine, Tongji University, Shanghai, China; d Medical College, Tongji University, Shanghai, China

**Keywords:** Stoma, Peristomal skin complications, Self-management, Evidence-based nursing

## Abstract

**Objective:**

To search, evaluate, and integrate evidence related to the self-management of peristomal skin complications in adults with intestinal stomas, in order to provide a scientific basis for the development of targeted and effective self-management support strategies for stoma patients.

**Methods:**

This study was conducted as an evidence summary following the reporting standards established by the Fudan University Center for Evidence-based Nursing. Following the '6S′ model, systematic searches were performed in both Chinese and English databases, relevant association websites, and guideline websites for literature on expert consensus, group standards, systematic reviews, clinical decisions, best practices, guidelines, evidence summaries, and randomized controlled trials pertaining to the self-management of peristomal skin complications in adults with intestinal stomas. The search period extended from the inception of the databases to February 17, 2025. Two researchers independently evaluated the quality of the literature and summarized evidence.

**Results:**

A total of 17 articles were included in this review, consisting of 7 guidelines, 1 group standard, 1 clinical decision, 4 systematic reviews, 2 expert consensus statements, and 2 randomized controlled trials. Through a comprehensive literature review, evidence extraction, and categorization, 27 pieces of evidence were summarized under three themes: medical management, role management and emotional management.

**Conclusions:**

This study systematically identified 27 evidence-based recommendations for self-management of adult with peristomal skin complications through three dimensions: medical management, role management, and emotional management. The evidence provides a robust, evidence-based foundation for healthcare providers, thereby making the self-management behaviors for peristomal skin complications in adults with intestinal stomas more scientific and targeted.

**Systematic review registration:**

This study was registered at Fudan University Center for the Evidence-based Nursing (Registration No. ES20257284).

## Introduction

Colorectal cancer (CRC) is one of the most common malignant tumors of the digestive system, characterized by high incidence and mortality rates.^1^ Studies show that it ranks third globally in incidence (10%) and second in mortality (9.4%) among all malignant tumors.^2^ Due to changes in lifestyle and dietary habits, the incidence and mortality rates of CRC are steadily increasing. The incidence is rising at an annual rate of 3.7% in urban males and 2.5% in urban females, with the age of onset gradually trending younger.^3^ According to statistics, CRC ranks among the top five most common cancers in China, and the country also has the highest number of new cases and deaths from CRC annually worldwide.^4^

Enterostomy is one of the effective surgical treatments for CRC.^5^ A segment of the patient's intestine is pulled out through the abdominal cavity, and the opening is everted and sutured to the abdominal wall, allowing the flow of feces to be redirected.^6^ The surgery enables the patient to excrete waste through the stoma on the abdominal wall, thereby serving as a substitute for anal defecation. Patients who undergo this procedure are required to live with an intestinal stoma. Statistical data indicate that the total number of CRC patients with intestinal stomas in China has exceeded 1 million, with approximately 100,000 new stoma patients added each year.^7^ According to statistics from the United Ostomy Associations of America, about 100,000 people undergo intestinal stoma surgery annually in the United States.^8^ This demonstrates that, with the increasing prevalence of CRC, the number of patients with intestinal stomas is also rising, making them a key population of concern for healthcare services both in China and globally. Although intestinal stoma surgery provides patients with a chance for survival and extends their life expectancy, it also alters their physiological structure. Due to patients' insufficient ability to manage stoma care, the incidence of stoma-related complications increases.^9^ Postoperative patients often experience various complications such as stoma edema, bleeding, prolapse, and stenosis, with peristomal skin complications (PSCs) being the most common, which cause significant physical and psychological distress, as well as financial burden for patients.^9^

PSCs refer to skin damage caused by various factors, which can be categorized into irritant dermatitis, mechanical injury, allergic dermatitis, infections and other diseases, skin hyperplasia, etc.^7^ They are the most common complications following stoma surgery. Studies indicate that 70% of stoma patients have experienced peristomal complications, with the incidence of PSCs ranging from 10% to 70%.^10^^,^^11^ PSCs not only threaten local tissue damage and cause pain but may also lead to systemic infections in patients, severely impacting their daily lives and imposing significant physical and psychological stress.^12^ Standardized and proper care is key to reducing these risks, addressing not only the skin issues but also serving as a core component in safeguarding patients' physical health, mental well-being, and quality of life.^11^^,^^12^ Additionally, while in-hospital nurses deliver specialized health education to patients with stomas, post-discharge education persists through various channels, including stoma knowledge lectures and support associations. Thus patients can access relevant care knowledge both during hospitalization and after discharge, thereby enhancing their ability to care for peristomal skin.^9^ Given the limited time that intestinal stoma patients spend receiving medical and nursing care in hospitals, and considering that most of their interactions with the stoma occur in community and home environments, it is essential for these patients to develop a certain level of self-management skills. This ability is crucial for coping with the changes and impacts that a stoma brings to their lives.

Self-management refers to individuals engaging in preventive or therapeutic health care activities under the guidance of healthcare professionals.^13^ Professor Kate Loring from the Stanford Patient Education Research Center proposed that self-management can be categorized into medical management, role management and emotional management.^14^ Medical management refers to the patient's ability to manage their own illness, such as taking medication, self-monitoring, and adhering to medical advice. Role management involves adapting to life changes brought about by chronic disease, adjusting and maintaining normal life roles. Emotional management entails handling negative emotions like anger, fear, and frustration arising from the illness, and cultivating a positive mindset to cope with the disease. In our study, medical management refers to comprehensive medical interventions, based on medical assessment, clinical judgment and evidence-based evidence, aimed at preventing and treating medical complications. Role management involves the adjustment and adaptation of patients' social roles and daily life functions after illness, such as balancing the needs of work, family, and social activities with stoma care, and re-establishing a sense of self-worth in social interactions. Emotional management focuses on the patient's psychological state regulation, including coping with negative emotions such as anxiety, depression, fear, and shame caused by stoma, and maintaining a positive and optimistic attitude towards life through effective emotional release and psychological support. Most stoma patients, after completing basic treatment during hospitalization, enter a chronic disease management process, making them the primary agents in self-managing their stomas and associated health conditions. For patients with stoma, the implementation of effective self-management is crucial to improving their quality of life, reducing the risk of complications, and enhancing treatment compliance. Studies have demonstrated that effective self-management enables patients to better cope with their conditions, reduces the incidence of stoma complications, lowers readmission rates, decreases medical costs, alleviates family burdens, and enhances quality of life.^15^ The Chronic Disease Self-Management Program (CDSMP), developed by Lorig KRH3, was initially designed for arthritis patients and later extended to the study of various chronic diseases, such as diabetes, hypertension, asthma, and arthritis.^16^ Subsequently, the program was implemented in Canada, the United Kingdom, Australia, and Europe. Research has demonstrated that self-management programs can significantly improve patients' health status and enhance their quality of life. Self-management is a form of empowerment, transforming patients from passive recipients of treatment into proactive managers of their own health. It directly impacts patients' physical comfort, psychological well-being, and overall quality of life. Receiving professional training and ongoing guidance from healthcare providers before and after stoma surgery, along with actively practicing self-management skills, is essential for every stoma patient to return to a fulfilling and normal life. However, the self-management ability of intestinal stoma patients remains at a moderate level, with most patients primarily relying on healthcare providers and exhibiting weak self-management awareness.^17^ This study systematically summarizes the relevant evidence on the self-management of PSCs in adults, aiming to provide evidence-based support for clinical healthcare providers in guiding patients' self-management, preventing PSCs, and maintaining long-term health and quality of life for adult stoma patients.

## Methods

### Problem establishment

The PIPOST model was used to establish evidence-based questions.^18^ The formed initial questions were as follows: Target population (P): Adult patients with intestinal stomas, including permanent and temporary stomas. Interventions (I): Patient's self-management. Professionals (P): Patients, healthcare providers. Outcome (O): Incidence of PSCs, awareness among healthcare providers, patients, and primary caregivers regarding the prevention and management of PSCs, patient quality of life, etc. Setting (S): Hospitals, households, communities. Type of evidence (T): Evidence summaries, guidelines, expert consensus, systematic reviews, group standards, clinical decision-making, best practices, and original studies closely related to the research topic.

### Literature searching

According to the “6S” model, searches were conducted following a top-down approach.^19^ The search was conducted in the following databases: CINAHL, Embase, Cochrane Library, Web of Science, PubMed, UpToDate, CNKI, Wanfang Database, VIP Database, and China Biology Medicine. Guideline sources comprised the NICE (National Institute for Health and Care Excellence) guideline repository and MedPulse. Relevant professional association websites included the World Council of Enterostomal Therapists (WCET), the Wound, Ostomy, and Continence Nurses Society (WOCN), and the International Ostomy Association. Search terms used were: “Stoma/Ostomies/Ostomy/Colostomy/Ileostomy/Enterostomy/peristomal/fecal diversion/ileal conduit∗/Conduit∗ ileal” “Dermatitis/Moisture-associated skin damage/MASD/Irritant dermatiti∗/fecal dermatiti∗/Dermatiti∗ Primary Irritant/Irritant Dermatiti∗ Primary/Primary Irritant Dermatiti∗/Dermatiti∗ Irritant” “Nursing/prevent/management/care”, combining free-text terms and MeSH, tailored to each database. The search time frame was from the database establishment to February 17, 2025. An example of the PubMed search strategy is provided in Table 1.

**Table 1:** Literature search strategy of PubMed.

#1 “Ostomies”[MeSH Terms]
#2 “Stoma”[Title/Abstract] “Ostomy”[Title/Abstract] OR “Colostomy” [Title/Abstract] OR “Ileostomy”[Title/Abstract] OR “Enterostomy” [Title/Abstract] OR “peristomal” [Title/Abstract] OR “fecal diversion” [Title/Abstract] OR “ileal conduit∗” [Title/Abstract] OR “Conduit∗,ileal” [Title/Abstract]
#3 #1 OR #2
#4 “Dermatitis”[MeSH Terms]
#5 “Moisture-associated skin damage”[Title/Abstract] OR “MASD”[Title/Abstract] OR “Irritant dermatiti∗”[Title/Abstract] OR “fecal dermatiti∗”[Title/Abstract] OR “Dermatiti∗ Primary Irritant”[Title/Abstract] OR “Irritant Dermatiti∗ Primary”[Title/Abstract] OR “Primary Irritant Dermatiti∗”[Title/Abstract] OR “Dermatiti∗ Irritant”[Title/Abstract]
#6 #4 OR #5
#7 “Nursing”[MeSH Terms]
#8 “prevent”[Title/Abstract] OR “management”[Title/Abstract] OR “care”[Title/Abstract]
#9 #7 OR #8
#10 “clinical practice guideline”[MeSH Terms] OR “review systematic”[MeSH Terms] OR “clinical trials randomized”[MeSH Terms]
#11 “Guidelines”[Title/Abstract] OR “consensus”[Title/Abstract] OR “evidence summar∗ ”[Title/Abstract] OR “systematic review”[Title/Abstract] OR “meta-analysis”[Title/Abstract] OR “randomiz∗”[Title/Abstract]
#12 #10 OR #11
#13 #3 AND #6 AND #9 AND #12

### Inclusion and exclusion criteria for literature

Inclusion criteria: ① The study included adult patients with intestinal stomas; ② The research content involved self-management of stoma patients with PSCs; ③ The literature types included evidence summaries, guidelines, expert consensus, systematic reviews, group standards, clinical decisions, best practices, and original studies closely related to the theme of this study; ④ The language was Chinese or English.

Exclusion criteria: ① Translated or interpreted versions of guidelines; ② Literature with missing contents or unable to obtain full text; ③ Literature that failed quality assessment.

### Literature quality evaluation methods

Four researchers with evidence-based professional knowledge independently evaluated the quality of included guidelines. For other types of literature included, two researchers firstly conducted independent evaluations according to the criteria. If there was any disagreement during the evaluation process, a third researcher joined the discussion until a consensus was reached. Guidelines were evaluated with the tool of AGREE II (Appraisal of Guidelines for Research and Evaluation II, AGREE II) for the assessment of healthcare guidelines.^20^ Clinical decisions were evaluated with CASE (Critical Appraisal for Summaries of Evidence, CASE).^21^ Systematic reviews were evaluated using AMSTAR (A Measure Tool to Assess Systematic Reviews, AMSTAR) for the assessment of systematic reviews.^22^ Expert consensus and group standards were evaluated using the corresponding evaluation tools from the JBI Center for Evidence-Based Health Care.^23^ Randomized controlled trials (RCTs) were evaluated using the Australian JBI Center for Evidence-Based Health Care (2016).^24^

### Evidence integration and grading

Two researchers independently extracted evidence on the prevention and care of PSCs in adult patients with intestinal stomas. Principles for evidence integration: evidence was synthesized and summarized based on logical relationships when content was complementary or consistent. Priority was given to high-quality and the most recently published evidence.^25^ If there were discrepancies in evidence integration, a third researcher was invited to participate in discussions until consensus was reached. After completing the evidence integration, the JBI Pre-Grading System for Evidence and Evidence Recommendation Levels (2014 Edition) was used to classify evidence into levels Level1 to 5.^26^

## Results

### Literature search results

**Fig. 1:**
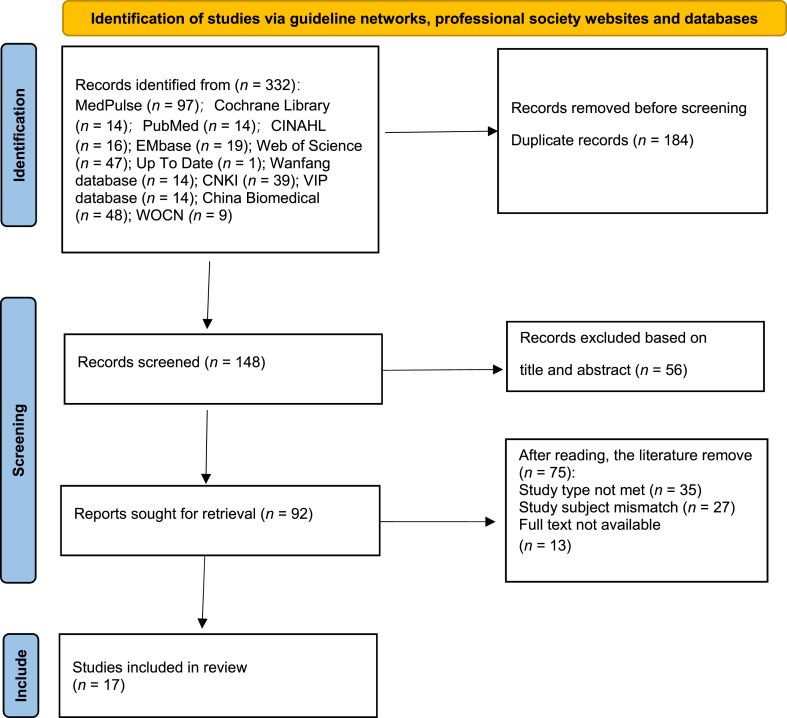
Illustrated the flowchart of literature screening process.

A preliminary search yielded 332 publications. After removing 184 duplicates and excluding 56 irrelevant records based on title and abstract screening, 92 records remained for initial review. Following full-text assessment, 35 articles were excluded due to mismatched study types, 27 for irrelevant research topics, and 13 for inaccessible full texts. Ultimately, 17 publications were included, comprising 7 guidelines,^27^, ^28^, ^29^, ^30^, ^31^, ^32^, ^33^ 4 systematic reviews,^10^^,^^34^, ^35^, ^36^ 2 expert consensus documents,^11^^,^^37^ 1 group standard,^38^ 1 clinical decision,^39^ and 2 RCTs.^40^^,^^41^ The included publications were published between 2015 and 2023, with 70% in English and originating from countries including China, the United States, the United Kingdom, and Germany. The literature search process was illustrated in Fig. 1, and the basic characteristics of the included studies were summarized in Table 2.

**Table 2:** Characteristic of included literature (*N* ​= ​17).

Included literature	Date of publication	Types of evidence	Source	Language	Country	Theme
China Stoma Management Collaboration Group, et al.^27^	2023	Guideline	MedPulse	Chinese	China	Classification and grading criteria of complications of stoma
Mathilde Aubert,et al.^28^	2023	Guideline	MedPulse	English	France	Management of adult intestinal stomas: The 2023 French guidelines
Chabal LO^29^	2021	Guideline	Pubmed	English	USA	Practice Implications from the WCET® International Ostomy Guidelin 2020
Hendren S, et al.^30^	2015	Guideline	Pubmed	English	USA	Clinical practice guidelines for ostomy surgery
WOCNS^31^	2018	Guideline	MedPulse	English	USA	Management of the adult patient with a fecal or urinary ostomy-an executive Summary
Readding L, et al.^32^	2016	Guideline	Pubmed	English	UK	Practical guidance for nurses caring for stoma patients with long-term conditions
Bradley R. Davis, M.D.^33^	2022	Guideline	MedPulse	English	USA	The American Society of Colon and Rectal Surgeons Clinical Practice Guidelines for Ostomy Surgery
Liu Yingge, et al. ^34^	2020	Systematic review	Wanfang	Chinese	China	Systematic review of factors affecting the occurrence of peristomal skin complications
Woo KY, et al. ^10^	2017	Systematic review	Pubmed	English	USA	Management of Moisture-Associated Skin Damage: A Scoping Review
Metcalf C ^35^	2018	Systematic review	Pubmed	English	UK	Managing moisture-associated skin damage in stoma care
Steinhagen E, et al.^36^	2017	Systematic review	Pubmed	English	USA	Intestinal Stomas-Postoperative Stoma Care and Peristomal Skin Complications
Dissemond J, et al.^11^	2021	Expert consensus	Pubmed	English	Germany	Moisture-associated skin damage (MASD): A best practice recommendation from Wund-D.A.CH. J Dtsch Dermatol Ges
Prinz A, et al.^37^	2015	Expert consensus	Pubmed	English	UK	Discharge planning for a patient with a new ostomy: best practice for clinicians
Chinese Nursing Association^38^	2020	Group standard	Chinese Nursing Association	Chinese	China	Adult ostomy care
Landmann RG, et al.^39^	2020	Clinical decision	Uptodate	English	USA	Ileostomy or colostomy care and complications
Chen Haiting ^40^	2021	RCT	Wanfang	Chinese	China	Evidence-based practice for the prevention and management of irritant dermatitis around the stoma in adult patients with enterostomy
Tan Yanzhu, et al.^41^	2019	RCT	Wanfang	Chinese	China	Evidence-based practice for the prevention and treatment of irritant dermatitis around stoma in patients with stoma

RCT, randomized controlled trial.

**Table 3:** Quality evaluation of included guidelines.

Included literature	Standardized scores in various domains (%)						Number of fields (greater than 60%)	Quality evaluation
	Scope and purpose	Stakeholder involvement	Rigor of development	Clarity of presentation	Applicablity	Editorial independence		
China Stoma Management Collaboration Group, et al.^27^	97.22%	91.67%	72.92%	80.56%	82.29%	100%	6	A
Mathilde Aubert, et al.^28^	97.22%	91.67%	72.92%	80.56%	82.29%	100%	6	A
Chabal LO^29^	94.44%	88.89%	60.42%	77.78%	62.5%	91.67%	6	A
Hendren S, et al^.30^	91.67%	77.78%	57.29%	81.94%	83.33%	91.67%	5	B
WOCNS ^31^	94.44%	88.89%	85.42%	88.89%	95.83%	83.33%	6	A
Readding et al.^32^	100%	50.69%	62.15%	62.15%	68.52%	100%	5	B
Bradley R. Davis, M.D.^33^	100%	89.58%	50.69%	68.52%	62.15%	91.67%	5	B

### Quality evaluation of included literature

(1) Quality evaluation results of the guidelines: a total of 7 guidelines were included.^27^, ^28^, ^29^, ^30^, ^31^, ^32^, ^33^ The standardized percentage and comprehensive evaluation results were shown in Table 3.

(2) Quality evaluation results of systematic reviews: a total of 4 systematic reviews were included. ^10^^,^^34^, ^35^, ^36^ The overall quality of the literature was high, and all systematic reviews were included.

(3) Quality evaluation results of expert consensus and group standards: a total of 2 expert consensus documents were included.^11^^,^^37^ And 1 group standard was also included.^38^

(4) Clinical decision-making evaluation result: one clinical decision-making document was included which was considered the highest level of evidence.^39^

(5) RCT quality evaluation results: a total of 2 RCTs were included.^40^^,^^41^ In the process of evaluating the quality of

RCTs published by Qin et al.,^41^ item 4 of the checklist from the Australian JBI Center for Evidence-Based Health Care was assessed as 'Unclear’, while the remaining items received a 'Yes' evaluation. Given that this study was relatively important, the evidence-based team decided to include it.

### Description and summary of evidence

After extracting, analyzing, comparing, and discussing the collected evidence, similar content was comprehensively integrated. Ultimately, 27 pieces of high-quality evidence were summarized across three key aspects: medical management, role management and emotional management, as illustrated in Table 4. Among the 16 pieces of evidence related to medical management, 11 pieces of evidence were strongly recommended as Level A evidence. These were summarized across the areas of stoma product management, skin care, basic monitoring, and complication intervention. In Evidence 5, it is recommended that postoperative patients should utilize skin protection products containing ceramides and select liquid film-forming agents, polyurethane films, zinc oxide ointment, or barrier paste to prevent or minimize direct skin contact with fluids and to protect the skin (1c). A multicenter, double-blind RCT conducted by Colwell et al. demonstrated that the incidence of PSCs was significantly lower in patients who used skin protection products containing ceramides after stoma surgery compared to those in the control group.^42^ In Evidence 6, the crusting technique was recommended for skin care. A RCT conducted by Park et al.^43^ compared the effects of standard peristomal skin care with the crusting technique on peristomal skin. The findings indicated that the crusting technique was more effective in reducing the incidence of peristomal skin erosion and tissue hyperplasia compared to standard care. Multiple studies recommended the use of standardized assessment tools, such as the Ostomy Skin Tool (OST), for the daily observation of stoma blood supply and the condition of the surrounding skin.^35^^,^^37^^,^^38^^,^^40^^,^^41^ These tools facilitated the assessment of the color, volume, consistency, and odor of the output, which aided in the timely detection of abnormalities in both the stoma and the surrounding skin, thereby monitoring the occurrence of PSCs (1c). Regarding role management, in Evidence 11: The WOCNS mentioned that patients with high stoma output should reduce the intake of hypertonic and hypotonic fluids, but specific intake range was not provided.^31^ Aubert et al.^28^ proposed an intake range of 500 mL–1000 mL. Since these 2 piece of evidence was recent publication, the two recommendations were combined. In Evidence 23: The WOCNS suggested that after discharge, patients should seek relevant knowledge through various avenues, including stoma clinics, home healthcare services, and telephone follow-ups.^31^ Davis et al.^33^ and Liu et al.^34^ proposed methods such as home visits and stoma associations to enhance post-discharge patient education and support. Therefore, integrating these two recommendations, it is advised that patients consult stoma therapists or other professionals trained in stoma care through various channels, including outpatient consultations, telephone follow-ups, home visits, and stoma support associations. This comprehensive approach will enhance their understanding of stoma care, gastrointestinal physiology, and anatomical functions (1a). In terms of emotional management, 4 pieces of evidence were identified. Evidence 25 illustrated that healthcare professionals who provided consultation regarding stoma-related knowledge facilitated patients' adaptation to their new appearance and effectively addressed the emotional challenges associated with anatomical changes and altered sutures (5b). Su et al.^44^ found that psychological care and health education delivered by medical staff significantly enhanced patients' long-term quality of life. Furthermore, as a robust external support system, companions and family members could offer increased companionship to patients and encourage participation in self-care (5b). Multiple studies confirmed that peer support education enhanced stoma acceptance and assisted patients in rebuilding their confidence for social reintegration.^44^, ^45^, ^46^, ^47^

**Table 4:** Evidence summary for the self management of PSCs in patients with a stoma.

Category	Evidence contents	Grade
1. Medical management	1. After measuring the size of the stoma, cut the opening of the stoma base to be 1–2 mm larger than the diameter of the stoma to avoid leakage and irritation of the skin around the stoma.^31^^,^^41^	1c
	2. Use a shaped pocket to reduce the gap between the stoma and prevent leakage.^41^	1c
	3. When the skin around the stoma is wrinkled and loose, the stoma is retracted or is leveled, convex stoma bags should be used, and high-volume stoma patients should choose wear-resistant bases.^10^^,^^29^, ^30^, ^31^^,^^35^	5b
	4. Use non-irritating skin protection film, stoma care powder or hydrocolloid dressing, etc., and use leak-proof paste, strip or ring if necessary; under the premise of ensuring the sealing of the base plate, brush off the excess stoma care powder, and then spray non-irritating liquid skin protection film.^36^^,^^11^	4a
	5. After operation, use skin protection products containing ceramide components, such as liquid film-forming agent, polyurethane film, zinc oxide ointment, leak-proof ointment, etc., to prevent or reduce the direct contact between skin and liquid, and to protect skin.^11^^,^^41^	1c
	6. Using crusting technique in prevention of peristomal skin complications.^41^	1c
	7. When changing the stoma bag, adhesive remover can be used for assistance .^29^^,^^31^	5b
	8. The following factors should be considered when choosing a stoma bag: stoma type and location, abdominal contour, lifestyle, patient vision and dexterity of hands. Choose a comfortable, odor-proof and skin-protecting stoma bag that can be worn for 3-7d.^10^^,^^32^^,^^39^^,^^41^	1c
	9. When the stoma base is whiten or rolled, it should be replaced as soon as possible and should be performed on an empty stomach in the morning.^40^	1c
	10. After surgery, the stoma was evaluated daily using the peristomal skin assessment tool (The Ostomy Skin Tool, OST) to observe the blood supply and surrounding skin of the stoma and find out any abnormal conditions around the stoma.^35^^,^^37^^,^^38^^,^^40^^,^^41^	1c
	11. The patient should observe the blood supply and surrounding skin of the stoma daily, as well as the color, quantity, nature and odor of the discharge.^40^^,^^41^	1c
	12. Do not use cream or oil-based skin care products before the stoma skin complications occur. Clean the skin around the stoma with warm water and soft paper towels or cloth.^11^^,^^41^	1c
	13. After complications occur around the skin of the stoma, firstly ask the patient whether there is leakage from the stoma bag and how long it has been worn to find out the cause and remove irritants on the skin surface in time; at the same time, observe the nature and quantity of feces, color and integrity of the skin around the stoma.^40^^,^^41^	1c
	14. For moist-related skin damage, use non-irritating skin protectants, stoma care powders, or hydrocolloid dressings; apply leak-proof ointment if necessary. For allergic contact dermatitis, immediately stop using stoma care products containing allergens and apply local medication. For mechanical skin injuries, use wound dressings; for adhesive-related skin damage, choose a stoma base without tape edges; for pressure injuries, remove the source of pressure.^38^	5b
	15. When the skin around the stoma is severely irritated, corticosteroids should be used for treatment, preferably in the form of rapid drying (spray).^28^^,^^40^^,^^41^	1c
	16. If PSCs occurs, seek medical advice immediately and follow the advice of professional nurses and surgeons.^28^	5b
2. Role management	17. Chewing food as much as possible, and take a light diet with little residue and no irritation as the main part. Avoid spicy and irritating foods and gas-producing foods, such as beans, onions, carbonated drinks, etc.^34^^,^^40^^,^^41^	1a
	18. For patients with high-volume colostomy, the intake of low-fiber liquids by mouth (such as water, tea, coffee, juice, and alcohol) should be limited to 500 ​mL to 1 ​L per day.^28^	5b
	19. The body weight of the patient should be controlled within ± 10% of the preoperative body weight.^41^	1c
	20. Extreme sports are strictly prohibited. Use a belt or add adhesive to keep the stoma bag sealed during exercise.^39^	5b
	21. Wear loose, comfortable clothing and carry extra stoma supplies when traveling to avoid exposing stoma bags and adhesives to extreme temperatures.^39^	5b
	22. Consultation can be conducted with medical staff on gastrointestinal physiology and anatomy.^28^^,^^31^^,^^41^	1c
	23. After the hospitalization, patients can consult with stoma therapists or other professionals who have received training in stoma knowledge through various forms such as outpatient consultation, telephone follow-up, home visit and stoma association.^31^^,^^33^^,^^34^	1a
3. Emotional management	24. Strengthen the patient's self-regulation ability by deep breathing, self-suggestion and listening to music.^33^	5b
	25. To adapt to the existing of stoma, health care providers can be consulted for guidance on stoma-related knowledge. This includes understanding new bowel patterns and effectively addressing various emotional issues associated with anatomical and suture changes.^39^	5b
	26. Peer education can be arranged to encourage patients to participate in self-care of stoma and enhance their acceptance of stoma.^38^	5b
	27. When patients feel distressed and painful, they should find someone to talk about it in time. Through relevant knowledge education and intervention, patients should face reality and do a good job with their families, giving them psychological support and guidance.^41^	1c

1a denotes systematic reviews of multiple randomized controlled trials; 1b denotes systematic reviews of multiple randomized controlled trials and other intervention studies; 2b denotes systematic reviews of multiple quasi-experimental studies and other low-quality intervention studies; 5a denotes systematic reviews of expert opinions; 5b denotes expert consensus; PSCs, peristomal skin complications.

## Discussion

### Self-management of PSCs in patients with a care: multifaceted strategies to prevention, monitoring, managing and patient education

PSCs are prevalent and have multifaceted impacts on patient's life. These conditions not only cause physical discomfort and infections but may also significantly affect psychological well-being, social interactions, and overall quality of life, potentially leading to anxiety and depression with severe consequences. Improving the quality of life for stoma patients necessitates the rapid enhancement of self-management skills to facilitate adaptation to daily life with a stoma. However, the current self-management capabilities of stoma patients remain at a moderate level,^17^ measures should be taken to promote the stoma patient's ability. Nurses play a crucial role as guides, educators, and supporters in peristomal skin self-management. While it is essential to assist patients in developing proper self-management awareness, the limited literature on PSCs management has resulted in ambiguous standards for patient care. The primary aim of this review was to identify and synthesize the best available evidence on self-management strategies for preventing and managing PSCs in adult CRC patients with a stoma. In line with this objective, a total of 17 high-quality sources—including guidelines, systematic reviews, expert consensus statements, and RCTs—were included. From these, 27 distinct evidence-based recommendations were extracted and categorized under three core themes: self-care, self-monitoring, and self-management. These findings provide a practical framework for guiding patients in developing effective self management behaviors and highlight specific interventions that can be integrated into medical management, role management, and emotional management. These findings provide evidence-based guidance for healthcare professionals to standardize scientific and targeted self-management practices for PSCs.

In chronic disease care, self-management emphasizes the patient's role as the primary steward of their health. Enterostomy not only alters patients' physiological excretion patterns but also initiates a chain reaction that affects medical management, social roles, and emotional well-being. Effective self-management necessitates coordinated efforts across these three dimensions to establish a holistic system that integrates physiological, social, and psychological aspects. While each dimension has distinct core objectives, they interpenetrate and mutually reinforce each other, collectively assisting patients in transitioning from medical management to life reconstruction.

#### Medical management

Medical management refers to the medical and nursing interventions that patients undertake to address their conditions. It focuses on the implementation of specific treatment plans and the management of physiological symptoms, forming the foundation of effective self-care.^14^ Through scientifically grounded and precise nursing practices, medical management aims to prevent complications, maintain stoma function, ensure physiological health, and facilitate patients' return to normal life. Evidence 1–16 systematically examine three critical dimensions: stoma care product management, skin care protocols, and complication management. Through a comprehensive literature review and evidence synthesis, this study highlights the pivotal role of disease management in the self-care practices of stoma patients. The analysis of Evidence 1–16 reveals that product selection and proper fitting, along with standardized replacement protocols, are fundamental for maintaining stoma stability and improving quality of life. Evidence 1, 3 and 7 demonstrate that the customized selection of stoma trays and pouches based on stoma type, size, skin condition, and patient activity levels significantly reduces leakage rates, while regular replacement routines minimize skin irritation. The development of innovative skin-protective stoma care bases and leak-proof technologies has emerged as a key focus in contemporary medical research. Patients requiring stoma care often favor advanced skin-protective stoma care bases and multi-layered leak-proof systems,^48^^,^^49^ which significantly reduce the risks of leakage. These solutions not only minimize PSCs but also enhance patients' health-related quality of life (HRQoL) by improving the management of liquid stool. Healthcare professionals should diligently monitor the skin condition surrounding stoma sites and promptly implement corrective measures, such as adjusting stoma care bases or administering anti-inflammatory medications, when abnormalities are detected. Regarding skin care, Evidence 2, 5, and 9 emphasize the importance of keeping the stoma area clean and dry, recommending tailored strategies for skin conditions such as irritant dermatitis and allergic dermatitis through the use of protective agents and leak-proof ointments. These approaches align with clinical principles of “prevention first, prevention and treatment combined.” The cleanliness and dryness of a stoma are critical factors that influence infection control, skin health, psychological well-being of patients, and overall quality of life.^50^^,^^51^ Therefore, standardized care and regular follow-ups are essential. Standardized care entails daily cleansing of the stoma area using mild, non-irritating cleansers while avoiding alcohol-based or fragranced products to prevent irritation. Regular follow-ups involve healthcare professionals periodically assessing the stoma and surrounding skin conditions, allowing for necessary adjustments to care plans. In cases of skin issues such as erythema or erosion, appropriate treatments should be administered based on the specific causes, which may include the application of skin protectants or antifungal medications, to promote healing and minimize complications. Evidence 4, 8, and 12 detail monitoring protocols for stoma color, shape, bowel movements, and vital signs, providing early warning mechanisms for potential complications. In the early postoperative phase, it is crucial to assess the stability of the stoma base to prevent morphological changes induced by increased abdominal pressure, such as coughing. Alterations in bowel patterns are associated with stoma complications, including anastomotic leakage and ischemia.^52^ Clinicians should meticulously document stool characteristics, as watery stools may indicate obstruction or infection, and monitor output volume, noting that an output exceeding 1.5 L per day warrants a dehydration alert. Furthermore, stoma-associated infections, such as parastomal hernias and abscesses, should be evaluated by monitoring vital signs, including temperature and local pain.^53^ In terms of complication management, Evidence 1, 6, 10, and 13–16 systematically address common postoperative complications such as stoma stenosis, prolapse, bleeding, and infection. These studies provide a comprehensive framework covering etiology analysis, clinical symptom identification, and specific management strategies, including conservative treatment and medical intervention criteria. Collectively, these evidence-based insights establish a medical management system that prioritizes prevention and precision intervention, which provides a solid theoretical basis and practical guidance for clinical medical staff to guide patients to carry out scientific and effective medical management. Therefore, healthcare professionals should educate patients and primary caregivers about the importance of disease management. This includes recognizing early signs of stoma complications and promptly contacting specialists or stoma clinics for follow-up care. Effective disease management can reduce unplanned readmissions and medical visits, thereby lowering social healthcare costs.^48^^,^^50^

#### Role management

Role management refers to patients' adaptation to changes in social roles, identity, and interpersonal relationships caused by disease.^14^ Enterostomy may disrupt the balance of patients' original social roles. The dimensions of role management focus on helping patients reposition their roles, restore social functions, and develop differentiated strategies for various role scenarios to prevent a decline in quality of life due to role loss. Evidence 17–23 summarizes aspects such as patient diet, activity, clothing, and stoma care. Evidence 17–19 provide dietary guidance for stoma patients. Research indicates that postoperative intestinal motility gradually recovers, with patients experiencing high-volume fluid output during the initial stoma phase. Concerns regarding the impact of different foods on stoma output necessitate expert dietary advice from stoma specialists.^54^ For patients unable to obtain sufficient nutrition postoperatively, nutritional supplements may serve as an alternative dietary source; however, their loose excretion can increase the risk of stoma-associated skin complications. Professional dietary counseling should emphasize food restrictions while also informing patients about available food options and dietary patterns. Studies demonstrate that post-discharge patients continue to require substantial nutritional information support, underscoring the need for continuous improvement of the continuum care service system.^55^ Evidence 22 and 23 indicates that patients need to actively seek knowledge and build support networks to adapt to stoma care. Solitano et al.^56^ found that patients who proactively acquired stoma-related knowledge and nursing skills had significantly lower risks of skin complications, such as dermatitis and leakage, compared to those receiving routine care. Role management addresses multiple dimensions, including diet, activity, clothing, and psychological adjustment, focusing on “role positioning” and “functional recovery” while developing differentiated strategies for different scenarios like diet, activity, and nursing. It is crucial to integrate stoma care into a part of life rather than allowing it to dominate, eliminate the stigma of disease, assist patients in reconstructing their identities, and remove the social and psychological barriers brought by illness, thereby achieving a fundamental improvement in quality of life. The enhanced knowledge of patients regarding stoma-related problems enables them to recognize warning signs of risk factors, propose appropriate preventive measures, improve adherence to recommended behaviors, and bolster their self-management capabilities.^54^^,^^55^ Consequently, healthcare professionals should prioritize health education to elevate patients' understanding of stoma care and related knowledge, thereby strengthening their self-management skills and awareness. The preoperative and postoperative phases during hospitalization are critical periods for disseminating knowledge. Patients can be educated through videos, brochures, and verbal instructions regarding risk factors for stoma complications, preventive measures, clinical manifestations, and nursing interventions. Given that stoma complications are chronic and progressive in nature, patients will remain in an extended self-management phase after discharge. Therefore, providing self-learning platforms—such as WeChat groups, official accounts, and mini-programs—becomes particularly essential.

#### Emotional management

Emotion Management refers to the strategies patients use to handle negative emotions such as fear, anger, frustration, depression, and uncertainty that arise from illness and lifestyle changes.^14^ Enterostomy triggers a range of negative emotions in patients, which may adversely affect compliance with medical management and the initiative to manage their roles effectively. The focus of emotion management is to assist patients in identifying their emotions, regulating their mental states, and facilitating a transformation from psychological acceptance to self-identity and proactive coping. Negative emotions primarily fall into three categories: shame, anxiety, and depression.^57^ Shame often arises from self-denial regarding the appearance of the stoma or fear of discrimination, particularly evident in the early postoperative stages, especially during initial encounters with the stoma or while changing the stoma bag. Anxiety generally stems from concerns about potential complications, which are prevalent during rehabilitation periods, such as worries about leakage impacting daily life or fears of stoma infections. Depression frequently results from prolonged caregiving pressures and fatigue associated with frequent stoma bag changes; without timely intervention, it can lead to persistent low mood. In the early postoperative period, acquiring knowledge about stoma can help dispel misconceptions. Additionally, techniques such as deep breathing, self-suggestion, and listening to music can enhance patients' self-regulation abilities, foster a positive mindset, and reduce self-criticism. During rehabilitation, patients striving to restore their social roles may experience anxiety.^33^ They are encouraged to express their concerns to family, friends, or peers to seek emotional support. When necessary, professional psychological counseling can help alleviate anxiety symptoms. Studies indicated that peer support education is effective in helping patients rebuild their confidence for reintegration into society. 44, 45, 46, 47^,^^58^ Even after adapting to their stoma, patients may still encounter emotional fluctuations. Consulting healthcare professionals for stoma-related knowledge and effectively addressing issues related to anatomical changes and altered sutures can contribute to achieving long-term psychological stability. Emotional management equips patients with the mental resilience needed to face challenges. A patient who maintains a calm mindset, feels accepted, and receives support is more likely to persist with complex daily care and actively explore and adapt to new lifestyles.

### Implications for nursing practice and research

Effective self-management of PSCs in stoma patients involves a comprehensive approach that encompasses not only medical aspects but also role adaptation and emotional resilience. Medical management focuses on preventive measures, such as proper stoma site selection, use of appropriate skin barriers, and regular skin inspection to detect early signs of irritation or infection. Role management addresses the patient's reintegration into social and occupational settings, emphasizing the importance of gradual exposure and support from healthcare professionals, family, and peers to overcome stigma and enhance self-esteem. Emotional management, on the other hand, involves recognizing and addressing the psychological impact of living with a stoma, including anxiety, depression, and body image issues. This can be achieved through counseling, peer support groups, and mindfulness-based interventions. By integrating these multifaceted strategies into a cohesive self-management program, healthcare professionals can empower stoma patients to take an active role in their care, leading to improved outcomes, enhanced quality of life, and reduced healthcare utilization. Furthermore, ongoing education and feedback mechanisms are essential to ensure the sustainability and adaptability of self-management practices over time.

### Limitations

Limitations of this study should be acknowledged. First, the included studies were predominantly conducted in developed regions, potentially limiting the generalizability of the recommendations to developing countries or regions with different healthcare resources and cultural contexts. Second, the quality of some included studies was moderate, with variations in sample sizes and methodological rigor, which might have affected the strength of the synthesized evidence. Future research should address these limitations by conducting multicenter studies in diverse cultural and socioeconomic settings, extending follow-up periods to evaluate long-term effects, and improving study designs to enhance the quality of evidence. Additionally, further exploration is needed to develop tailored self-management interventions that consider individual patient characteristics, such as age, comorbidities, and educational level, to maximize the applicability and effectiveness of the recommendations. Despite these limitations, the 27 evidence-based recommendations derived from this study offer valuable and practical guidance for clinical practice, empowering clinical nurses and caregivers to better support stoma patients in achieving optimal self-management and improving their overall well-being.

## Conclusions

This evidence-based study systematically identified 27 evidence-based recommendations for self-management of adult with PSCs through three dimensions: medical management, role management, and emotional management. The comprehensive overview aims to provide evidence-based guidance for clinical nurses and caregivers to reduce PSCs and improve quality of life for stoma patients. The findings of this study have significant implications for both clinical practice and future research. For clinical nurses and caregivers, these evidence-based recommendations offer a structured framework to support adult patients with PSCs in their self-management journey. Furthermore, the study highlights the importance of a holistic approach to stoma care, encompassing not only medical but also role and emotional aspects, which can contribute to a more patient-centered care model. These findings provide a foundation for further exploration and refinement of self-management interventions tailored to the specific needs of stoma patients.

## CRediT authorship contribution statement

**Jiayu Qin:** Conceptualization, Methodology, Writing – Original Draft, Funding Acquisition. **Lijun Han:** Methodology, Writing – Original Draft. **Guifen Lv:** Conceptualization, Methodology. **Yongmei You:** Supervision, Writing – Review & Editing. **Huiren Zhuang:** Supervision, Writing – Review & Editing. **Lili Ma:** Conceptualization, Methodology, Writing – Original Draft, Supervision, Writing – Review & Editing. All authors have read and approved the final manuscript.

## Ethics statement

Not required.

## Data availability statement

The authors confirm that the data supporting the findings of this study are available within the article.

## Declaration of generative AI and AI-assisted technologies in the writing process

No AI tools/services were used during the preparation of this work.

## Funding

This study is funded by Angel training program of Shanghai East Hospital (Grant No. DFTS-2218). The funders had no role in considering the study design or in the collection, analysis, interpretation of data, writing of the report, or decision to submit the article for publication.

## Declaration of competing interest

The authors declare no conflict of interest.
